# Epidemiology of dengue fever in Ethiopia: Insights from a systematic review and meta-analysis

**DOI:** 10.1016/j.parepi.2026.e00523

**Published:** 2026-06-27

**Authors:** Mohamed Omar Osman, Dejene Hailu Kassa, Taye Gari, Embialle Mengistie Beyene

**Affiliations:** a Department of Public Health, College of Medicine and Health Science, Hawassa University, Hawassa, Ethiopia; b Department of Public Health, Institute of Health Science, Jigjiga University, Jigjiga, Ethiopia

**Keywords:** Dengue fever, Prevalence, Determinants, Ethiopia, Systematic review and meta-analysis, Mosquito-borne disease, Public health

## Abstract

**Background:**

Dengue fever is an emerging mosquito-borne viral disease with increasing reports from several regions in Ethiopia. However, national-level pooled evidence on its prevalence and determinants remains limited on dengue fever.

**Objectives:**

This systematic review and meta-analysis aimed to estimate the pooled prevalence of dengue fever and identify determinants among patients in Ethiopia.

**Methods:**

This systematic review followed PRISMA guidelines and registered the protocol in PROSPERO (CRD420251180312). Electronic databases and university repositories were searched. A random-effect model was used to estimate pooled prevalence and pooled odds ratios (ORs). Heterogeneity was assessed using the I^2^ statistic and Cochran's Q test. Subgroup analyses were conducted based on study design and diagnostic methods.

**Results:**

Thirteen studies met the inclusion criteria in this meta-analysis. Eight cross-sectional studies were included in the pooled prevalence analysis. The systematic review and meta-analysis found that the dengue fever pooled prevalence in Ethiopia was 25% (95% CI: 23–27), with no significant heterogeneity across the studies (I^2^ = 0.00%), while the pooled estimate of the prevalence was statistically significant (*p* < 0.001). The subgroup analysis showed a higher prevalence of dengue cases before the year 2020 (35%) compared to after 2020 (16%). Determinant analyses showed that not wearing long-sleeved clothes, not using a mosquito net, the presence of open water containers, and not spraying insecticides in the last six months were significant factors associated with dengue infection, with heterogeneity being high (I^2^ > 75% of studies, *p* < 0.001).

**Conclusion:**

Dengue fever is an important public health concern in Ethiopia, particularly in outbreak-prone and urban neighborhoods. However, pooled estimates should be interpreted cautiously due to methodological heterogeneity. The current study identified that behavioral and environmental factors significantly influence the risk of dengue fever infection. These findings demonstrate the importance of targeted public health interventions (community education on preventive practices and enhanced mosquito control strategies).

## 1 Introduction

Dengue fever is a serious mosquito-borne illness that is becoming increasingly common in many parts of the world, including Ethiopia, that is quickly spreading and is a global public health problem, mostly in tropical and subtropical areas (Bhatt et al., 2013; Harapan et al., 2020).

The dengue virus (DENV), which is responsible for all categories of DF infections in the world, has four distinct but related serotypes (DENV1–4), which belong to the family *Flaviviridae* (Murugesan and Manoharan, 2020).

Dengue fever is a viral infection that can often be identified with clinical signs that include thrombocytopenia, leucopenia, and elevated liver function in addition to fever, headache, myalgia/arthralgia, and skin flushing/rash (Rehman et al., 2022; Tsai et al., 2013).

Dengue fever is misdiagnosed as malaria because it is a complex illness with numerous clinical signs and is often misinterpreted or misdiagnosed due to the African pandemic, resulting in an overestimation of 75% of cases (Amarasinghe et al., 2011a, Amarasinghe et al., 2011b). In Africa, particularly Ethiopia, the spread of infectious pathogens like Ebola, malaria, and COVID-19 is likely to complicate control and response programs (Wainaina et al., 2022). The diagnosis can be performed by detecting the virus, viral nucleic acids, antigens, anti-DENV antibodies, or combinations of these techniques (WHO, 2009). In the early stages of the disease (seven or less than 7 days after onset of illness), DENV infection can be diagnosed from serum, plasma, circulating blood cells, or other tissues (CDC, 2019). There is no specific treatment for dengue, but the timely diagnosis of dengue cases, identification of warning signs for severe dengue, and appropriate clinical management are key elements of care to prevent the progression to severe dengue and deaths (Fagbami et al., 1977; Kalimuddin et al., 2025).

The incidence of dengue has grown dramatically around the world in recent decades, with cases reported to WHO increasing from 505,430 cases in 2000 to 5.2 million in 2019 (WHO, 2024). Despite being a neglected tropical disease, DF is a significant issue on the African continent, with 22 countries reporting sporadic cases or outbreaks between 1960 and 2010, primarily from DEV2. (Amarasinghe et al., 2011a, Amarasinghe et al., 2011b; Fagbami et al., 1977).

The first laboratory-confirmed outbreak of dengue fever in Ethiopia was reported in 2013, with more than 12,000 dengue fever cases documented (WHO, 2018). Since this time, outbreaks have been confirmed in the northern and eastern parts of the country; the following year, in 2014, outbreaks occurred again in Dire Dawa (Mohammed Yusuf et al., 2019), as well as in the southeastern part of the country in Godey Town, Somali Region, and Adaar Woreda in Afar Region, which is located in northern Ethiopia, and outbreaks have since occurred every year in Godey Town (Ahmed and Salah, 2016; Degife et al., 2019b; Health—Ethiopia., M. o, 2014). Additional evidence of DF in northern Ethiopia was detected in the Tigray Region and Amhara Region via a serological cross-sectional study of febrile patients in these regions (Ferede et al., 2018a) and the Borena southern Oromia region (Geleta, 2019).

Even though DENV infection would be transmitted all year round in Ethiopia, the risk of contracting it in the country is the most significant during and immediately after the rainy season, which runs from June to August (B. N. Gobena et al., 2025). The close contact with DF patients, non-use of bed nets, and the presence of stagnant water around the village were identified as risk factors for contracting DF in Ethiopia (Degife et al., 2019a).

Evidence suggests that updating the pooled prevalence and determinants of dengue fever is essential for developing an action plan and implementing the necessary interventions to prevent tropical diseases. While individual, persuasive published studies have been conducted in Ethiopia to present data regarding the prevalence and determinants of dengue fever. However, magnitude and determinants have not been well documented nationally in a pooled way that makes scientific sense in this case. This systematic review and meta-analysis provide updated pooled prevalence and identify determinants of dengue fever to assist health planners, policymakers, and the community in reducing the effects of dengue fever. Therefore, this systematic review and meta-analysis aimed to estimate the pooled prevalence of dengue fever and identify determinants among patients in Ethiopia.

## 2 Methods and materials

Protocol and registration: The protocol titled “Epidemiology of Dengue Fever in Ethiopia: Insight from Systematic Review and Meta-Analysis” was registered with the International Prospective Register of Systematic Reviews (PROSPERO) at the University of York Centre for Reviews and Dissemination (https://www.crd.york.ac.uk/) with the registration number (CRD420251180312). This was conducted in accordance with the Preferred Reporting Items for Systematic Reviews and Meta-Analysis Protocols (PRISMA-P) checklist guideline (Additional File 2) (Page et al., 2021).

## 3 Search strategy

A comprehensive literature search of biomedical electronic databases such as PubMed/Medline, Scopus, HINARI, African Journal Online (AJOL), Science Direct, Google Scholar, and Ethiopian university repositories. The full Boolean search strategy is provided in the supplementary file of Additional File 1. The search Medical Subject Headings (MeSH terms) include: (“Dengue” OR “Dengue Fever”) AND (“Prevalence” OR “Seroprevalence” OR “incidence”) AND (“determinants” OR “risk factors” OR “associated factors”) AND (“Ethiopia”). The literature search was done from database inception throughout June 2025.

## 4 Eligibility criteria

This systematic review and meta-analysis included studies that reported the prevalence and/or determinants of dengue fever among patients in Ethiopia. Studies were eligible if they (1) were conducted in Ethiopia, (2) reported a laboratory-confirmed dengue infection, (3) used cross-sectional and case-control study designs with a response rate of at least 80%, and (4) were published in English. Grey literature (dissertations and theses from Ethiopian university repositories) was included if it met the inclusion criteria and provided sufficient methodological details. Non-peer-reviewed reports without full methodological descriptions were excluded.

This systematic review used the PICO (Population, Intervention, Comparison, and Outcomes) in the search engine:

**PICO: P (Population):** Individuals in Ethiopia who were participants in studies investigating dengue fever.

**I (intervention/exposure):** Exposure to potential determinants/risk factors for dengue fever.

**C (Comparison):** Individuals without exposure to the identified risk factors (reference groups with the included studies).

**O (Outcome):** Prevalence/magnitude of dengue fever and its associated determinants/factors.

## 5 Data Extraction/Abstraction

Four authors independently reviewed the titles and abstracts of the included articles and reviewed the full text of the selected articles according to the eligibility criteria. Moreover, discrepancies between authors were resolved through discussion and consensus. Data extractions were conducted independently by four authors (MO, DH, TG, and EM). We solved the disagreement through verification and further discussion. The following data were extracted for analysis: author, publication year, setting of the study, sample size, number of patients with dengue fever, diagnostic method used, case definition, and adjusted odds ratio for determinants with 95% confidence intervals.

## 6 Operationalization of outcome measures

The primary outcome of this study was laboratory-confirmed dengue infection. Studies using IgM antibody or NS1 antigen or PCR were considered indicative of acute or recent infection. Studies using IgG seropositivity were considered reflective of past exposure. The main outcome assessed was the prevalence and determinants of dengue fever in Ethiopia. The prevalence of dengue fever was determined by dividing the number of individuals with dengue fever by the total number of participants in the study, then multiplying by 100 to obtain a proportion. To analyze factors associated with dengue fever, data were gathered using the two-by-two table method from various studies, and the adjusted odds ratio (OR) was calculated to identify the relationship between each independent variable and the dependent variable. Only cross-sectional studies were included in the pooled prevalence meta-analysis, and case-control studies were excluded from prevalence pooling and were used solely for determinant analysis.

## 7 Synthesis of Results/Statistical Analysis

Information about the studies was summarized using Microsoft Excel and then exported to STATA version 13 software (Stata-Corp. LP, College Station, TX, USA). The estimated pooled prevalence of dengue fever and its determinants was calculated using a random-effects meta-analysis model with a constrained maximum likelihood technique, taking into account the heterogeneity across the studies. The forest plot was utilized in this systematic review and meta-analysis to display the pooled estimate with a 95% confidence interval (95% CI). The pooled estimate with a 95% confidence interval (95% CI) was displayed using a forest plot in this systematic review and meta-analysis. The results of the I-squared (I^2^) test were used to assess statistical heterogeneity. The proportion attributable to study variability is shown by the I^2^ statistic. The heterogeneity of the included studies was interpreted as follows, with I^2^ statistic values ranging from 0% to 100%: low heterogeneity for <50%, moderate heterogeneity for 50%–75%, and high heterogeneity for greater than or equal to 75% (Chiappelli et al., 2010). For meta-analysis with a minimum of 10 studies, publication bias was determined based on the visual appraisal of the funnel plot (Borenstein et al., 2010).

## 8 Sub-Group Analyses

Subgroup analyses were performed based on the year of publication in which these studies were published and based on diagnostic methods (RT-PCR vs. ELISA) to minimize epidemiological misclassification and assess heterogeneity arising from diagnostic variability.

## 9 Quality assessment for studies

Joanna Briggs Institute Meta-Analysis of Statistics Assessment and Review Instrument (JBI-MAStARI) (Munn et al., 2014) was applied for critical appraisal of incorporated studies prior to data extraction. Random selection of the study sample, a comprehensible definition of the criteria for the inclusion of the sample in the study, identification and addressing of confounding factors, use of objective criteria to appraise the outcome of interest, reliable measurement of the outcome variable, and use of appropriate statistical analysis methods were included in the appraisal tool. After quality assessment, studies that scored five and above out of nine criteria set by the JBI for prevalence studies were included in this review and meta-analysis. All included studies in the final review scored between 7 and 9 on the JBI quality assessment checklist, indicating high methodological quality.

## 10 Results

### 10.1 Search result and description of studies

**Fig. 1:**
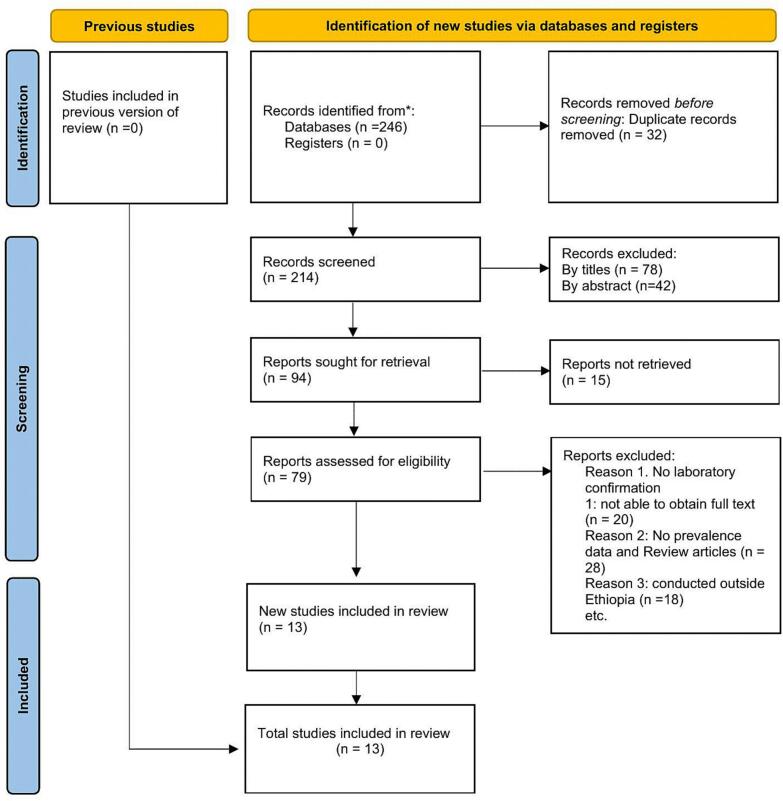
Updated PRISMA 2020 flow diagram for systematic reviews.

A total of 246 articles were retrieved for this systematic review and meta-analysis. About 32 of these were eliminated for duplication of records and again by unrelated titles 78 and abstract 42. Following the evaluation of 94 reports sought for retrieval, 15 reports were not retrieved, and 66 were further eliminated because the particular outcome variable (the prevalence was not reported) was able to obtain full access, was conducted outside Ethiopia, and reports were not retrieved. Lastly, 13 articles were ultimately incorporated into this comprehensive systematic review and meta-analysis **(**Fig. 1**).**

### 10.2 Characteristics of included studies

In this systematic review and meta-analysis, 13 studies with 3321 participants were included to estimate the prevalence and determinants of dengue fever among patients in Ethiopia. Of these, 8 studies were cross-sectional studies that contributed to the prevalence meta-analysis, and 5 case-control studies were included only in the determinants analysis (Ahmed and Salah, 2016; Akelew et al., 2022; Biru et al., 2020; Degife et al., 2019a; Eshetu et al., 2020; Ferede et al., 2018b; Geleta, and medicine, r. i. t, 2019; Bikila Negesa Gobena et al., 2025; Gutu et al., 2021; Mesfin et al., 2022; Shimelis et al., 2023; Sisay et al., 2022; Woyessa et al., 2014) studies **(**Table 1**).**

**Table 1:** Characteristics of included studies.

Authors	Study Design	Population	Region	n	N	JBI score
Akelew et al. (2022)	Cross-sectional	Acute febrile	Amhara	28	200	7
Ferede et al., 2018a, Ferede et al., 2018b	Cross-sectional	Acute febrile	Amhara/Tigray	200	600	8
Geleta, 2019	Case control	Suspected	Dire Dawa		180	8
Sisay et al. (2022)	Cross sectional	Suspected	Dire Dawa	13	60	8
Woyessa et al. (2014)	Cross sectional	Suspected	Dire Dawa	50	88	7
Degife et al., 2019a, Degife et al., 2019b	Case control	Case control	Dire Dawa		210	7
Geleta, and medicine, r. i. t, 2019	Cross-sectional	Acute febrile	Oromia	160	519	8
Gobena et al. (2025)	Case control	Case control	Oromia		150	7
Eshetu et al. (2020)	Cross-sectional	Acute febrile	SERS	175	529	8
Shimelis et al. (2023)	Cross sectional	Acute febrile	Sidama	9	407	8
Ahmed and Salah, 2016	Cross-sectional	Suspected	Somali	33	57	8
Gutu et al. (2021)	Case-control	Suspected	Somali		150	7
Mesfin et al. (2022)	Case-control	Suspected	Somali		171	8
**NB:** Case-control studies (*n* = 5) were used only for determinant analyses						

Table 1**.** Characteristics of included studies.

### 10.3 Proportion of dengue fever in Ethiopia

**Fig. 2:**
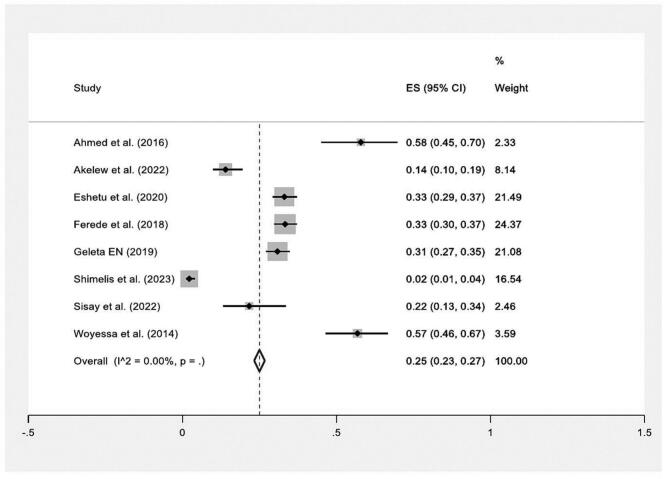
Pooled prevalence of dengue fever in Ethiopia.

In the descriptive analysis, which included 8 articles, the estimated pooled prevalence of dengue fever among patients attending health facilities in Ethiopia was 25% (95% CI: 23, 27). The I-square test showed that there was no heterogeneity among the included studies in the prevalence estimate studies (I^2^ = 0.00%) and the pooled prevalence estimate was statistically significant (p- < 0.001) **(**Fig. 2**).**

### 10.4 Sub-group analysis

**Fig. 3:**
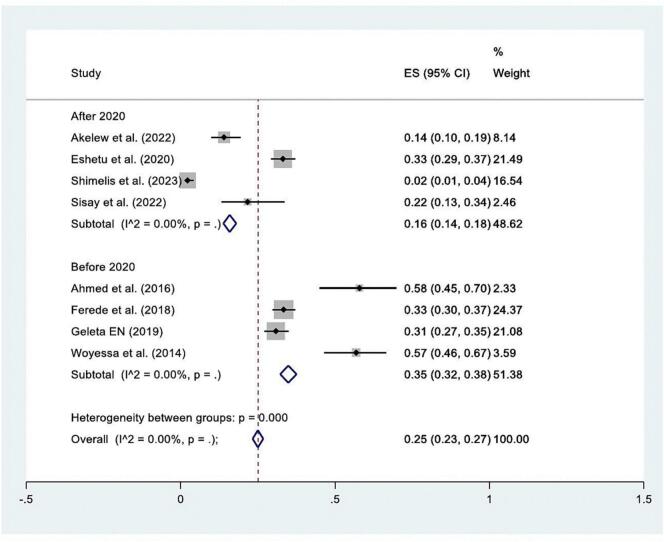
Sub-group analysis of the included studies based on published year.

Subgroup analysis was based on year of publication (before 2020 and after 2020), with 2020 selected as a cut-off to explore the potential effect of the COVID-19 pandemic on dengue surveillance and reporting. The highest prevalence was reported before the year 2020, which was 35% (95% CI: 32, 38), while the year after 2020 was 16% (95% CI: 14, 18) (I^2^ = 0.00%, *p* < 0.001) **(**Fig. 3**).**

**Fig. 4:**
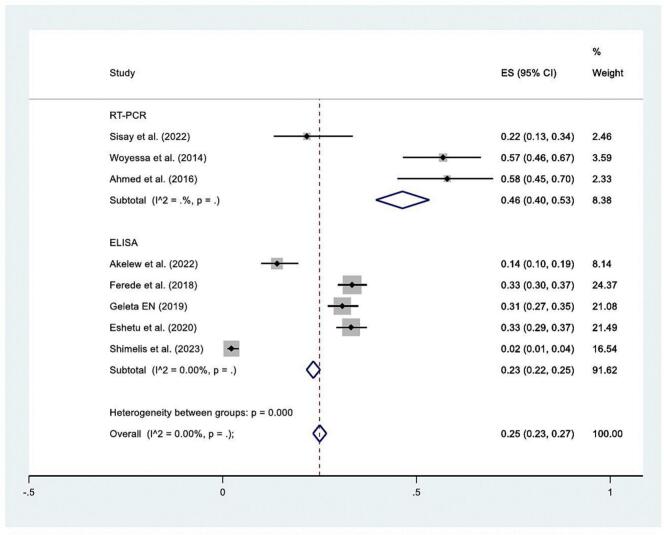
Subgroup analysis of the included studies based on diagnostic type.

In another subgroup analysis, the forest plot shows a pooled estimate derived from studies using two diagnostic methods (RT-PCR and ELISA) for dengue fever. When stratified by diagnostic method, RT-PCR studies reported a higher pooled prevalence of 46% (95% CI: 40–53%), while ELISA-based studies showed a lower pooled prevalence of 23% (95% CI: 22–25%) with between-group heterogeneity, which was significant (p < 0.001), showing that prevalence estimates differ meaningfully by diagnostic method rather than by random variation **(**Fig. 4**)**.

**Fig. 5:**
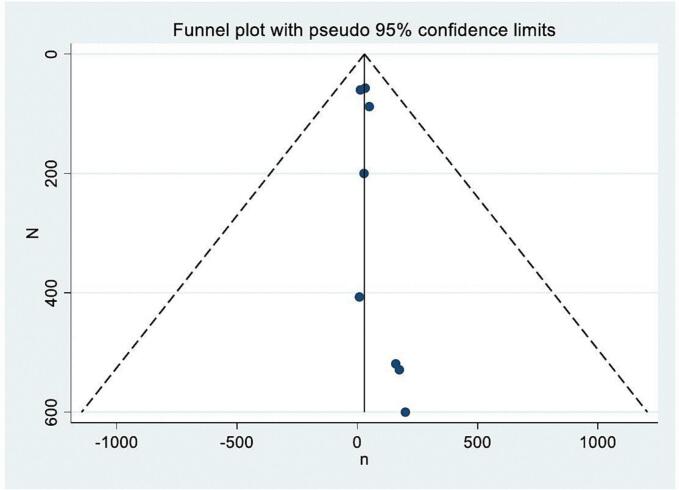
The funnel plot showing publication bias.

The funnel plot shows symmetry, with fewer small studies on the left side, suggesting no considerable publication bias of the study **(**Fig. 5**).**

### 10.5 Factors associated with dengue fever

**Fig. 6:**
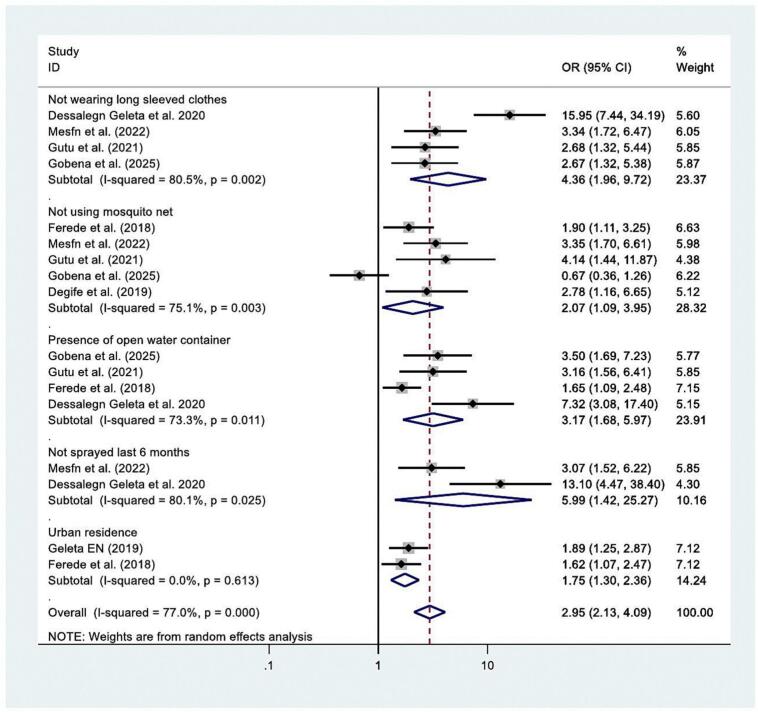
Forest plot of pooled odds ratios for factors associated with dengue fever in Ethiopia.

This systematic review and meta-analysis pooled five variables; each derived from different studies. Four of them showed significance, associations with dengue fever, though all analyses demonstrating substantial heterogeneity (I^2^ range: 73.3%–80.5%), namely not wearing long-sleeved clothes, not using a mosquito net, the presence of an open water container, and not spraying in the last 6 months. Not wearing sleeved clothes was one of the pooled significant variables; those who don't wear sleeved clothes were 4 times more likely to get infected compared to those who wear them (AOR = 4.3, 95% CI: 1.9–9.7, *p*-value <0.002). Another significant variable was not using a mosquito net; study participants who didn't use a mosquito net were 2 times more likely to be infected compared to their compatriots (AOR = 2.07, 95% CI: 1.09–3.95, p-value <0.003). Having an open water container in the household was 3 times more likely to get infected with dengue fever compared to their opposite (AOR = 3.1, 95% CI: 1.6–5.9, p-value <0.011). Lastly, not spraying in the last 6 months in the house was significantly associated with dengue fever; households that did not spray in the last 6 months were 5.9 times more likely to develop dengue fever compared to those who sprayed in the last 6 months (AOR = 5.9, 95% CI: 1.4–25.2, p-value <0.025). Heterogeneity tests presented an indication of high heterogeneity among studies that assessed determinant factors, which include not wearing sleeved clothes (80.5%), not using a mosquito net (75.1%), having an open water container (73.3%), and not spraying in the last 6 months (80.1%) and the overall heterogeneity was (I^2^ > 75% studies *p* < 0.001).) **(**Fig. 6**).**

## 11 Discussion

This systematic review and meta-analysis reports dengue fever pooled prevalence and analyzes predictors in Ethiopia. The overall prevalence was found to be 25% (95% CI: 23–27%), which indicates a significant burden, as elsewhere in the rest of the world in other dengue-endemic regions. Through research. For instance, in Southeast Asia, higher rates of prevalence have been reported, with estimated seroprevalence rates for Indonesia and Thailand ranging from 30% to 60% in fever patients (Shepard et al., 2013). In Brazil, a seroprevalence study found that IgG positivity rates were over 50%, especially in crowded cities like Rio de Janeiro and São Paulo (Teixeira et al., 2002). Currently, dengue fever is regarded as an emerging public health concern, despite being underreported in Africa. A multicounty review study reported that dengue outbreaks occurred in 22 African countries, highlighting the continent's vulnerability and the difficulties caused by inadequate surveillance systems and misdiagnosis with other febrile illnesses such as malaria (A. Amarasinghe et al., 2011). A study done in Kenya showed that more than 61.2% of febrile patients had IgG/IgM positivity for the dengue virus (Lim et al., 2020). This similarity in prevalence levels increases the probability that endemic dengue transmission is already occurring in Ethiopia and possibly other East African countries on a scale equivalent to areas that previously had established dengue endemicity.

In subgroup analysis, studies were characterized into two categories; studies done prior to 2020 reported a higher dengue prevalence (35%) than those done after 2020 (16%). This temporal difference may be due to multiple factors; these include disruption in the dengue surveillance system during the COVID-19 pandemic, diversion of diagnostic funding away from surveillance of febrile illnesses, reduced healthcare-seeking behavior, and potential reduction in transmission due to pandemic-related movement restrictions (Din et al., 2021). However, without data from longitudinal, consistent surveillance systems, this hypothesis requires further investigation.

The current analysis also identified a number of important factors that are significantly associated with a higher risk of dengue; one of these is not wearing long-sleeved clothes, the presence of an open water container, and not spraying the mosquito in the last 6 months. These factors found in this study align with regional studies and multi-country WHO reports that *Aedes aegypti* breeding and dengue transmission are significantly influenced by stagnant water in the surroundings and poor environmental sanitation (WHO, 2017). According to a multi-country analysis study, it was identified that poor water storage practices and limited household-level vector control were significant variables in dengue outbreaks (Sarker et al., 2024). Wearing protective clothing and household spraying considerably reduced the transmission of dengue fever in neighborhoods in Brazil (Teixeira et al., 2002).

Globally, climate change and urbanization are increasingly recognized as critical drivers of dengue infection transmission (Bhatt et al., 2013; Gubler et al., 2001). Studies have shown that climate variability significantly influences Aedes mosquito distribution and dengue virus transmission patterns, mainly in arid and semi-arid climate climatically vulnerable regions. (E. Abbasi, 2025a, Abbasi, 2025b; Mordecai et al., 2019; Ryan et al., 2019). New geographic areas and the expansion of Aedes mosquitoes, facilitated by climate change and human mobility, have contributed to the spread of arboviral diseases in transboundary areas (Ebrahim Abbasi, 2025b; Kraemer et al., 2015; Messina et al., 2019).

While much of the evidence on climate-driven transmission originates from studies conducted outside Ethiopia, the biological and ecological mechanisms underlying climate-sensitive transmission are broadly applicable across geographic settings, including Ethiopia, where heterogeneous climate zones and rapid urbanization may create favorable conditions for vector spread and virus transmission (Ebrahim Abbasi, 2025a; E. Abbasi, 2025b; Abbasi and Moemenbellah-Fard, 2025; WHO, 2022).

Therefore, future studies in Ethiopia should incorporate climate and environmental data to better understand spatial and temporal patterns of dengue risk and to inform models for prediction of outbreak early warning systems (Abbasi, 2025c, Abbasi, 2025d, Abbasi, 2025e).

## 12 Limitations

A major limitation of this meta-analysis is that there is heterogeneity observed, particularly in determinant analysis. A potential source of the heterogeneity was variation in the study design, diagnostic methods, population differences, and inconsistent adjustment for the confounders.

## 13 Conclusion

This systematic review and meta-analysis found that dengue fever has a substantial burden in Ethiopia. Although not as high as levels reported in hyperendemic regions of Southeast Asia and Latin America, it reflects a significant and unacknowledged public health problem in this country. While findings suggest increasing case reports of dengue across regions, most of the included studies were conducted during outbreaks and in health facilities; hence, the pooled prevalence may not yet represent the stable condition of the country's transmission pattern.

Moreover, the study identified modifiable determinants with dengue, including not wearing long-sleeved clothes, the presence of an open water container, and not spraying for mosquitoes in the last 6 months. These factors indicate absences in community-level vector control initiatives and individual preventive practices. The evidence suggests that the burden of dengue fever in Ethiopia can be considerably decreased with relatively feasible interventions. The results of this meta-analysis suggest that in order to improve prompt detection and response, dengue fever should be incorporated into national surveillance systems.

To properly manage and reduce dengue transmission in Ethiopia, it is imperative to raise public awareness of personal protection, enhance community-level vector control, strengthen the diagnostic capabilities of the health facilities, and encourage context-specific, especially community knowledge, attitude, and prevention research. Finally, improving laboratory-based surveillance, integrating risk monitoring which is climate-informed, and strengthening outbreak readiness are essential for effective dengue control in Ethiopia.

## CRediT authorship contribution statement

**Mohamed Omar Osman:** Writing – review & editing, Writing – original draft, Visualization, Validation, Supervision, Software, Resources, Project administration, Methodology, Investigation, Formal analysis, Data curation, Conceptualization. **Dejene Hailu Kassa:** Writing – review & editing, Writing – original draft, Visualization, Validation, Supervision, Software, Resources, Project administration, Methodology, Investigation, Formal analysis, Data curation, Conceptualization. **Taye Gari:** Writing – review & editing, Writing – original draft, Visualization, Validation, Supervision, Software, Resources, Project administration, Methodology, Investigation, Formal analysis, Data curation, Conceptualization. **Embialle Mengistie Beyene:** Writing – review & editing, Writing – original draft, Visualization, Validation, Supervision, Software, Resources, Project administration, Methodology, Investigation, Formal analysis, Data curation, Conceptualization.

## Consent for Publication

Not applicable.

## Ethics approval and consent to participate

Not applicable: Because the data included in this manuscript is publicly available.

## Funding

Not applicable.

## Declaration of competing interest

The authors declare no competing interests in this manuscript.

## Appendix A Supplementary data

**Supplementary material 1: Additional file 1:** Supplementary file showing search strategies from database on dengue fever in Ethiopia.

**Supplementary material 2: Additional file 2:** PRISMA checklist for systematic review and meta-analysis.
